# Data in support of three phase partitioning of zingibain, a milk-clotting enzyme from *Zingiber officinale* Roscoe rhizomes

**DOI:** 10.1016/j.dib.2016.01.014

**Published:** 2016-01-16

**Authors:** Mohammed Gagaoua, Kahina Hafid, Naouel Hoggas

**Affiliations:** Equipe Maquav, INATAA, Université Frères Mentouri Constantine, Route de Ain El-Bey, 25000 Constantine, Algeria

**Keywords:** Three Phase Partitioning, Zingibain, Purification

## Abstract

This paper describes data related to a research article titled “Three Phase Partitioning of zingibain, a milk-clotting enzyme from *Zingiber officinale* Roscoe rhizomes” (Gagaoua et al., 2015) [1]. Zingibain (EC 3.4.22.67), is a coagulant cysteine protease and a meat tenderizer agent that have been reported to produce satisfactory final products in dairy and meat technology, respectively. Zingibains were exclusively purified using chromatographic techniques with very low yield purification. This paper includes data of the effect of temperature, usual salts and organic solvents on the efficiency of the three phase partitioning (TPP) system. Also it includes data of the kinetic activity characterization of the purified zingibain using TPP purification approach.

**Specifications Table**

**Table:** 

Subject area	Biology, Chemistry
More specific subject area	Protein purification, Three Phase Partitioning, Enzymology
Type of data	Table, text file, figure
How data was acquired	Extraction, Three Phase Partitioning, Electrophoresis, Zymography, Enzyme kinetic
Data format	Raw and processed data
Experimental factors	Protein extraction, purification factors, pH, temperature, thermal and storage stability, molecular weight and purity.
Experimental features	Zingibain was first recovered using an optimized Three Phase Partitioning protocol and then characterized.
Data source location	Maquav Team, INATAA Institute, Constantine, Algeria
Data accessibility	Data is with this article

**Value of the data**

• Data for high purity purification of zingibain using Three Phase Partitioning (TPP) system are given.
• The protocol and data described provide optimized parameters for TPP tool for an efficient recovery of zingibain from ginger rhizomes.
• Values of optimal factors affecting TPP system are provided.
• Kinetic characterizations of the purified zingibain are given for the first time.

## 1 Data

**Fig. 1:**
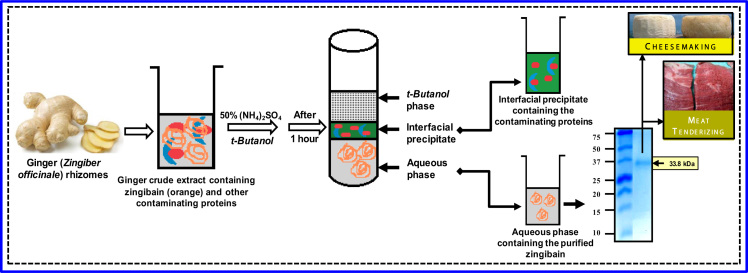
Three phase partitioning of zingibain from *Zingiber officinale* roscoe rhizomes and their use in food industry for cheesemaking and meat tenderizing. The purified zingibain (33.8 kDa assessed by SDS–PAGE) was found in the aqueous phase giving a 14.91 purification fold with 215% activity recovery.

**Fig. 2:**
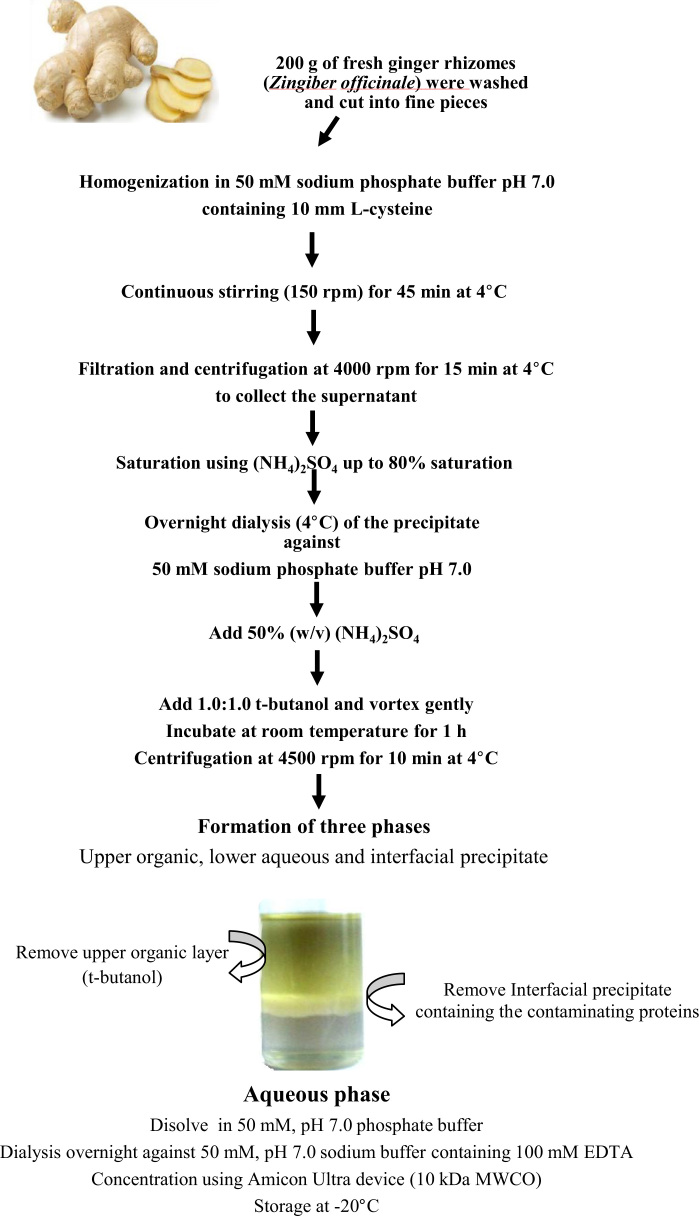
Diagrammatic representation of the workflow employed to purify zingibain from fresh *Zingiber officinale* Roscoe rhizomes using three phase partitioning (TPP) system.

Zingibain (EC 3.4.22.67), is an extensively used protease in food industry for cheese-making or meat tenderization, and therefore its fast and efficient purification is highly required. In the present work, we provide data of the optimal parameters for an efficient purification tool, the Three Phase Partitioning system for high purity and recovery yield of zingibain. A table containing the effect of usual salts and organic solvents on partitioning of zingibain by TPP is given (Table 1). In addition a flow sheet of the workflow employed to purify zingibain from fresh *Zingiber officinale* Roscoe rhizomes followed by the Kinetics (Michaelis-Menten and Lineweaver-Burk plot) of the purified zingibain are shared (Fig. 1, Fig. 2).

## 2 Experimental design, materials and methods

### 2.1 Zingibain extraction and TPP purification

Fresh fully ripened ginger rhizomes (*Z. officinale* roscoe) were purchased from a local market in Khenchela region (North–East) of Algeria. As summarized in Fig. 2, an approximate of 200 g of the rhizomes were washed and cut into fine pieces (~2.5 mm thickness) before homogenization in a blender with 450 mL of 50 mM sodium phosphate buffer (pH 7.0) containing 10 mM L-cysteine. The obtained homogenate was then left to stand under a continuous stirring (150 rpm) for 45 min at 4 °C before filtration through a double-layered cheesecloth. The insoluble residual debris was discarded and the supernatant was filtered through Whatman paper no. 1. The obtained ginger juice was spun in a centrifuge at 4000 rpm for 15 min at 4 °C to collect the supernatant, which was concentrated using ammonium sulfate up to 80% saturation. After an overnight dialysis (membrane molecular weight cut off (MWCO): 14 kDa) of the precipitate against two changes of 5 L of 50 mM sodium phosphate buffer, pH 7.0 at 4 °C, the crude clarified and dialyzed enzyme extract was subjected to TPP purification system.

**Fig. 3:**
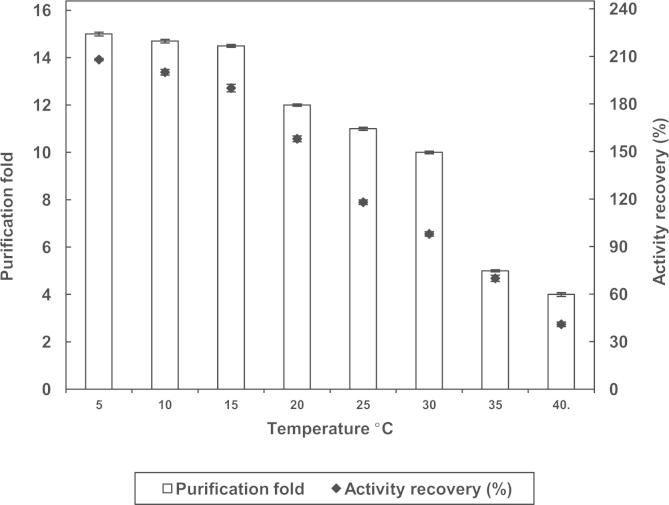
Influence of temperature on the degree of purification and activity recovery of *Zingiber officinale* zingibain. Ammonium sulfate (50% w/v) was added to the crude extract (2 mL containing 61.68 U). The pH of the medium was adjusted to pH 7.0. This was followed by addition of t-butanol in a ratio of 1.0:1.0 (v/v).

TPP experiments were carried out as described in our paper published in International Journal of Biological Macromolecules 73 (2015) 245–252 [1]. Prior the TPP process, different usual salts (Ammonium sulfate, potassium sulfate, sodium sulfate, sodium chloride, magnesium sulfate, and potassium chloride) and organic solvents (t-butanol, n-butanol, n-propanol, Iso propanol, petroleum ether and ethanol) were assayed as shown in Table 1. The results showed that *tert*-butanol and ammonium sulfate give the best results for the TPP process. After the optimization of the parameters (pH, ammonium sulfate, *tert*-butanol and temperature) affecting the TPP process, three repetitions were conducted to confirm the overall results using 50% ammonium sulfate, 1.0:1.0 ratio crude extract to t-butanol at a pH of 7.0 and allowed to stand for 1 h at a temperature of 5 °C. The temperature of the system was varied from 5 °C to 40 °C to determine the best value leading to obtain the best partitioning behavior of the system (Fig. 3). Afterwards, the mixture was centrifuged at 4500 rpm for 10 min to facilitate the separation of the three phases. The upper t-butanol phase was removed by a Pasteur pipette. The lower aqueous phase and the interfacial phase were separated carefully and analyzed for enzyme activity and protein content. The interfacial precipitate was dissolved in 50 mM, pH 7.0 sodium phosphate buffer. The aqueous phase always containing the higher zingibain activity was collected, dissolved in 50 mM, pH 7.0 sodium phosphate buffer, dialyzed overnight against the same buffer containing 100 mM EDTA and concentrated by ultrafiltration using Amicon Ultra device with a 10 kDa MWCO. The concentrated enzyme was stored at +4 °C or −20 °C until use for further characterization studies in order to determine the general biochemical properties.

### 2.2 Enzyme characterization

The protein concentration was quantified by the dye binding method of Bradford [2] using the Bio-Rad Protein Assay (Bio-Rad Laboratories Inc.). The protein content was calculated from the bovine serum albumin (BSA) standard curve.

Zingibain activity was assayed at 40 °C using bovine casein (1% w/v) as substrate in a 50 mM sodium phosphate buffer pH 7.0 containing 250 mM EDTA and 1 mM DTT at enzyme concentrations of 0.20 mg/mL. The hydrolyzing activity of zingibain was based on the method described by Kunitz using denatured bovine casein as a substrate [3], [4]. In activity measurements, 0.12 mL enzyme was incubated at 37±1 °C for 15 min prior to the assay. The reaction was stopped by the addition of 1.8 mL of 5% trichloroacetic acid (TCA). For the blank, the substrate was added after the enzyme was first inactivated by TCA. The resulting precipitate was removed by centrifugation at 4500 rpm for 15 min, and the absorbance of TCA soluble peptides in the supernatant was measured at 280 nm. One unit of activity is defined as the amount of enzyme that increases the absorbance by 0.01 min^−1^ under given assay conditions.

**Fig. 4:**
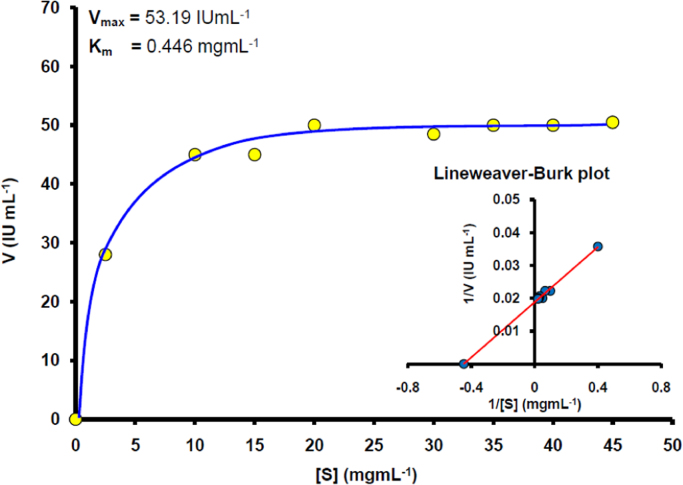
Kinetics (Michaelis-Menten and Lineweaver-Burk plot) of the purified zingibain from ginger rhizomes using Three Phase Partitioning.

*K*m and *V*max of the purified zingibain were determined by measuring the enzymatic activity using different concentration of bovine casein substrate (Fig. 4). The activity was measured under the standard assay conditions as discussed above. Michaelis-Menten and Lineweaver-Burk (Reciprocal) plots were acquired using SigmaPlot 12 software to determine *km* and *Vmax*.

## Conflict of interest

The authors declare they have no conflict of interest.

## Figures and Tables

**Table 1:** Effect of usual salts and organic solvents on partitioning of zingibain by TPP.

Salt	Relative enzyme activity (%)	Organic solvent	Relative enzyme activity (%)
Ammonium sulfate	100	t-butanol	100
Potassium sulfate	42	n-butanol	68
Sodium sulfate	31	n-propanol	62
Sodium chloride	32	Iso propanol	45
Magnesium sulfate	28	Petroleum ether	15
Potassium chloride	29	Ethanol	11
