# Supplementary material for: Data in support of three phase partitioning of zingibain, a milk-clotting enzyme from Zingiber officinale Roscoe rhizomes
Source: Data Brief. 2016 Jan 16;6:634–9. doi: 10.1016/j.dib.2016.01.014 (PMC4735474; doi:10.1016/j.dib.2016.01.014)

Author declaration

We wish to confirm that there are no known conflicts of interest associated with this publication and there has been no significant financial support for this work that could have influenced its outcome.

We confirm that the manuscript has been read and approved by all named authors and that there are no other persons who satisfied the criteria for authorship but are not listed. We further confirm that the order of authors listed in the manuscript has been approved by all of us.

We understand that the Corresponding Author is the sole contact for the Editorial process (including Editorial Manager and direct communications with the office). He is responsible for communicating with the other authors about progress, submissions of revisions and final approval of proofs. We confirm that we have provided current, correct email addresses which are accessible by the Corresponding Author (Dr. Mohammed GAGAOUA) and which have been configured to accept email from: gmber2001@yahoo.fr or mgagaoua@inataa.org

On behalf of my co-authors Naouel HOGGAS and Kahina HAFID

Dr. Mohammed GAGAOUA

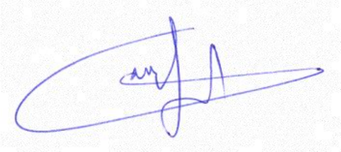

Supplement: Supplementary file 1 — Supplementary material [file mmc1.pdf]
